# Microbial Profiles of Meat at Different Stages of the Distribution Chain from the Abattoir to Retail Outlets

**DOI:** 10.3390/ijerph20031986

**Published:** 2023-01-21

**Authors:** Zikhona Theodora Rani, Lindokuhle Christopher Mhlongo, Arno Hugo

**Affiliations:** 1 Department of Animal and Poultry Science, School of Agricultural, Earth and Environmental Sciences, University of KwaZulu-Natal, P Bag X01, Scottsville 3209, South Africa; 2 Department of Animal Science, University of the Free State, P. O. Box 339, Bloemfontein 9300, South Africa

**Keywords:** bacterial count, *E. coli*, loading, off-loading, *S. aureus*, storage duration

## Abstract

Meat has been found to be a prime vehicle for the dissemination of foodborne pathogens to humans worldwide. Microbial meat contaminants can cause food-borne diseases in humans. The threat to consumers by microbial meat contaminants necessitates the studying of meat microbial loads to prevent potential illnesses in consumers. Studies investigating the meat microbial loads in South Africa are limited. The objective of this study was to compare microbial contamination of different meat types from low-throughput (LTA) and high-throughput abattoirs (HTA) at three stages of the distribution chain from abattoir to retail outlets. Beef, pork, and mutton (n = 216) carcasses were sampled: during the loading process at the abattoirs, when off-loading at the supply points and during marketing. All samples were subjected to total bacterial count (TBC), coliform count (CC), presumptive *Escherichia coli* (*E. coli*) (PEC) and *Staphylococcus aureus (S. aureus*) detection. In mutton, TBC dominated at loading, CC was similar across distribution chain stages, PEC was the predominant microbial contaminant at the offloading stage at the HTA, but TBC was affected at loading, CC was similar across distribution chain stages, PEC was affected at loading, and *S. aureus* was affected at the display stage at the LTAs. In beef, TBC had similar levels at loading; CC and PEC dominated at the display stage for the HTAs. However, TBC was affected at the display stage; CC was similar across stages; PEC was affected at the offloading stage at the LTAs. In pork, higher contamination levels were discovered at the display stage, CC dominated at the loading stage, with PEC detected at offloading at the HTAs but TBC, CC, PEC and *S. aureus* were similar across stages at the LTAs. TBC, CC and PEC were affected by the storage period and meat supplier to meat shop distance whereas distance affected the TBC, CC and PEC. Meat supplier to meat shop distance negatively correlated with meat distribution chain stage but positively correlated with TBC, CC and PEC such as temperature. Temperature positively correlated with meat distribution chain stage and shop class. Meat distribution chain stage was negatively correlated with storage period, TBC, CC and PEC but positively correlated with shop class. Shop class negatively correlated with storage period, TBC, CC and PEC. Storage period positively correlated with TB, CC and PEC. TBC and meat type positively correlated with CC and PEC. CC positively correlated with PEC but negatively correlated with *S. aureus* such as PEC. In conclusion, mutton, pork and beef meat are susceptible to microbial contamination at distribution chain stages in abattoirs.

## 1. Introduction

Meat is an excellent source of protein in the human diet; however, it could be highly susceptible to microbial contamination [1,2]. Meat contains approximately 75% water and 21% nitrogenous compounds (19% proteins and 1.5% non-protein nitrogen compounds). Meat chemical composition predisposes the meat to contamination if appropriate hygienic practices are not followed [3,4]. Hence, the main objective of the meat handlers should be to ensure a low initial number of microorganisms in meat because the meat industry intends to produce meat with a low number of microbes as reasonably possible, to maximize shelf-life and minimize the occurrence of organisms associated with food-borne illness [5,6,7].

Past studies [8,9] reported the importance of controlling the growth of microorganisms in meat to avoid outbreaks of food-borne diseases, which have occurred previously in other countries. In South Africa, abattoirs that deliver meat to retailers for consumers to purchase are governed by the Meat Safety Act of 2000 [10]. Meat processors in these abattoirs adopted a range of protocols and regulations which ensure that the meat produced is of high quality and safe for consumption, and that chances of microbial contamination are minimized. However, when meat is distributed from the abattoir to the supply points, it is out of the direct control of the abattoir of origin. Meat inspection is typically only carried out at the abattoir, yet the distribution stage is the most critical stage during which the quality of the meat could easily be compromised. Such could allow food-borne pathogens and more spoilage bacteria to enter the distribution chain.

Previous results [11,12] identified contamination from pathogenic microorganisms as the most serious meat safety issue, as it causes immediate consumer health problems. Food-borne illnesses caused about 3000 deaths in the United States of America and the cost of foodborne disease is estimated to exceed USD 5 billion per year [13]. In 2015, a report by the World Health Organization [14] documented that 600 million foodborne illnesses were responsible for 420,000 deaths. A recent government-commission report on the South African meat inspection system estimates the cost to the economy in medical, legal, other expenses, and absenteeism from work and school resulting from food-borne diseases to amount to billions of Rands annually and further recommends a review of the meat safety risk management systems and quality assurance practices (Department of Health [DoH], Department of Trade and Industry [DTI], and Department of Agriculture, Forestry and Fisheries [15]. However, to date, there is no scientific data available in South Africa for the microbiological quality of meat along the value chain.

In South Africa, meat inspection audits at the abattoir only cover the visual inspection, there are no microbiological tests that are done. Pathogens such as *S. aureus* and *E. coli* cannot be detected with the naked eye [16]. Safe meat production, processing, and distribution in South Africa are managed by two main regulatory entities, (DAFF) and the DoH [17]. However, these public meat monitoring agents do not have a meat Microbiological Monitoring Programme. Therefore, an improvement to cater to meat safety along the food chain that incorporates microbiological assessment of meat at the abattoir after slaughter and at retail using conventional methods is encouraged. Even though similar studies were conducted in different years elsewhere, it is important to see the microbial load of the meat in South Africa since there was no such study in the country. Therefore, the objective of this study was to compare microbial contamination at different stages of the distribution chain, from abattoirs to retail outlets. The hypothesis tested was that there are no differences in the microbial profiles of red meat at different stages of the distribution chain, from abattoir to retail outlets.

## 2. Materials and Methods

### 2.1. Study Site

This study was conducted in two selected abattoirs, the Nxuba Municipality local municipality and the Buffalo City Metropolitan Municipality (BCB) in the Eastern Cape Province of South Africa. The abattoirs were classified into two major categories, which are the low-throughput abattoir (Nxuba: LTA) and the high-throughput abattoir (BCB: HTA). Both abattoirs are governed by the Meat Safety Act [10] and the South African Meat Industry Company [18]. The high-throughput abattoir (HTA) is equipped with modern technology and slaughters up to 1000 livestock units per day with different slaughter floors for each species, while the low-throughput abattoir (LTA) operates with low-tech technology and slaughters less than 40 animal units per day. All animal species use the same slaughter floor in both abattoirs. After slaughter, the HTA chills the carcasses for approximately 16 to 20 h at 5 and 7 °C before distribution to retail outlets. In the LTA they do not have chiller rooms; therefore, carcasses are distributed to the retail outlets an hour to 2 h after slaughter. Three retail shops supplied by the HTA and a butchery supplied by the LTA abattoir were also selected and visited after distribution.

### 2.2. Sampling Procedure

A total of 216 swab samples were collected from beef, pork and mutton carcasses at three different selected stages: loading, off-loading and display (n = 12 for each species at each stage) from both abattoirs. Sampling was carried out aseptically by swabbing the muscular surface of each carcass with sterile cotton swabs moistened in sterile 0.1% buffered peptone water (BPW). The number of carcasses to be sampled per day was calculated according to Food Safety and Inspection Service (FSIS) Directive 6420.2 and the Livestock Carcass Examination. Carcasses were randomly selected at the abattoir before loading the trucks for delivery to the retail shops. An area of 16 cm^2^ marked with a sterile frame of 4 cm × 4 cm on each side of the carcass was rubbed for 30 s and swabs were transferred to a screw-capped McCartney bottle containing 10 mL of sterile 0.1% BPW.

Different sites from each carcass were used in each of the three species. The sampling sites were selected according to ISO 17604, (2003) and European Union (EU) Directive 2001/471/EC guidelines [10] For beef carcasses, the brisket, neck, perineal and medial areas were used. For mutton carcasses, neck, brisket, flank, and perineal areas were used. For pork carcasses, the area between the elbow and the midline cut, the area below the level of the axis joint, and the base of the tail towards the hock were used. All carcasses were loaded in the same truck and followed to the supply points, with the distance from the abattoir having been recorded. Bar codes were used to trace carcasses for identification. Swab samples were then collected from the same carcasses during off-loading at the supply points. Appointments were made with the butchery managers for purchasing meat samples from the same carcasses at the display outlets, where an area of 6.25 cm^2^ demarcated using a sterile frame of 2.5 cm × 2.5 cm was swabbed. The swab samples were stored in an icebox at 4 °C to prevent microbial growth and transported on the same day to the University of Free State for laboratory analysis. The packages were kept intact until they were aseptically opened in the laboratory for examination.

### 2.3. Microbiological Analyses

Samples were analysed immediately upon arrival at the laboratory. Total bacteria count (general bacteria), coliform count (related to hygiene and indicator for pathogens), *Escherichia coli* (Gram-negative pathogen) and *Staphylococcus aureus* (Gram-positive pathogen) were determined.

#### 2.3.1. Total Bacteria Count

Tenfold serial dilutions of the samples were prepared in 0.1% BPW up to 10^–8^ after which 1 mL aliquots of the respective dilutions were gently mixed as pour plates with 15–20 mL of standard plate count agar (SPCA, Oxoid CM463). After setting, the agar plates were incubated at 32 °C for 48 h. Colonies were counted using a digital colony counter (CJ Labs, RSA). Counts per cm^2^ were estimated depending on the area of the template used.

#### 2.3.2. Coliform and *E. coli* Counts

The sample dilutions were cultivated as pour plates with violet red bile agar with MUG (VRBM, Oxoid CM0978). The plates were incubated at 37 °C for 24 h. Coliforms were enumerated as the purple-red colonies, while generic *E. coli* were enumerated as the number of fluorescent colonies after subjecting the VRBM agar plates to ultraviolet radiation at 364 nm. Counts per cm^2^ were estimated depending on the area of the template used.

#### 2.3.3. *Staphylococcus aureus*

Sample dilutions were spread-plated on Baird Parker Agar (Oxoid, CM025), a selective medium for the isolation and counting of coagulase-positive staphylococci, followed by aerobic incubation at 37 °C for 48 h as described by [18]. Presumptive colonies were counted, and population per cm^2^ was estimated depending on the area of the template used. The presumptive colonies were confirmed using the API^®^ Staph Kit (Biomerieux) according to the manufacturer’s instructions.

### 2.4. Statistical Analysis

Data on microbiological counts were first transformed to log (base 10). The Generalized Linear Model of Statistical Analysis System [19] was used to determine the effect of meat type, distribution stage, shop class, storage period, distance and temperature on the microbial contaminants. A Pearson correlation analysis was carried out between distance, temperature, meat type, stage, shop class, storage period and the response variables (TBC, CC, presumptive *E. coli* and *S. aureus*). Mean separation was conducted using the student *t*-test.

## 4. Results and Discussion

### 4.1. Microbial Counts as Affected by the Abattoir, Meat Type and Distribution Stage

**Figure 1:**
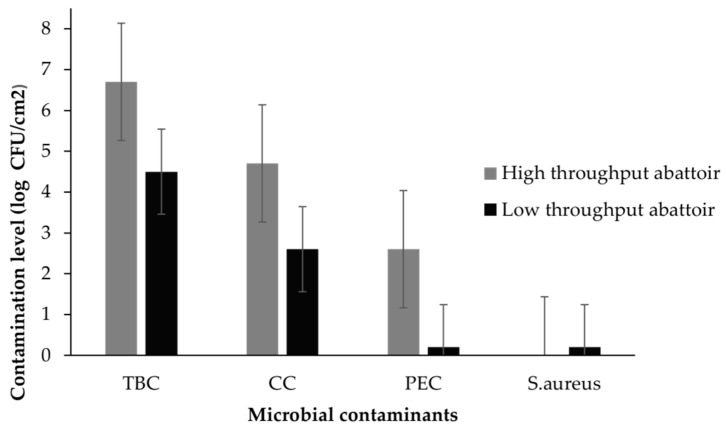
Contamination levels in beef, pork and mutton carcasses in a commercial and a communal abattoir (TBC = total bacteria count; CC = coliform count; PEC = presumptive *E. coli*) (n = 108). Error bars indicate average ± standard deviation of meat microbial contaminants.

Microbial contamination levels of beef, pork, and mutton carcasses before transportation from the HTA and LTA abattoirs are shown in Figure 1. There were no significant differences (*p* > 0.05) in meat microbial contaminants between LTAs and HTAs (Figure 1). *E. coli* (G -ve) was the most predominant pathogen, followed by *S. aureus* (G +ve). These results are similar to past results [20]. The TBC, CC and *E. coli* were higher in the HTA (6.9 log CFU/cm^2^; 4.7 log CFU/cm^2^ and 2.6 log CFU/cm^2^) than in the LTA (4.5 log CFU/cm^2^, 2.6 log CFU/cm^2^ and 0.2 log CFU/cm^2^), respectively. 

The higher microbial load in carcasses from the HTA indicates poor storage conditions during transportation and the handling of carcasses. Such could also be attributed to the large number of animals slaughtered (up to 1000 livestock) units per day for the HTA, with an appreciably large number of workers handling the meat on the floor compared to the LTA which slaughters less than 40 animals per day. Although it was observed that meat handlers wore protective clothing or gloves when handling the meat, there were no sanitizers used for decontamination after touching each carcass while it was handled by five or more different people. Therefore, cross-contamination between meat handlers’ responsible for packing the carcasses in chiller rooms at the HTA or dressing them for transportation may occur. These findings are similar to the past reports [21,22,23], therefore, contributing to bacterial contamination of meat. Furthermore, cross-contamination may have also occurred from mixing different types of meat from different species, whereas at the LTA, they only slaughter one type of species per day without having to mix beef, pork, or mutton carcasses at the same time [24]. In most cases at the abattoirs, differences in microbial load are mostly due to a lack of good processing practices, good handling practices and sanitary standard operating procedures along the production chain [9,25]. Nevertheless, *S. aureus* was not detected at all in carcasses that were collected from the HTA but were found at the LTA abattoir. The presence of *S. aureus* on carcasses from LTA could be attributed to the poor sanitary quality of abattoirs [26]. These results agree with a past study [27], although the levels detected were found to be lower (0.2 log CFU/cm^2^) than in the current study.

Results on the microbial contamination levels per meat type at different stages from the abattoir to the supply points are shown in Table 1. Significant differences among different stages for beef, pork and mutton on total bacteria count, coliform count and *E. coli* were observed. Beef carcasses from the HTA had higher levels of microbial contamination for all tested bacteria, followed by mutton and pork. Similar results were reported in a previous study [28], where beef showed higher general viable counts than mutton and pork. A study [29] highlighted that poorly organized farms-to-table production chains and poor standard sanitary operational procedures practised by the abattoir personnel, including poor personnel hygiene, are some of the risk factors that contribute to the high microbial load.

The levels of TBC among beef, mutton and pork from the HTA were 7.7, 7.2 and 5.9 log CFU/cm^2^, respectively, and coliform counts were 5.8, 4.2 and 3.9 log CFU/cm^2^, respectively, for the loading stage at the abattoir. The high levels of total bacteria count in this study follow previous studies [18,30,31,32]. However, according to a previous study [12], compared to the European Microbiological Standards for meat, these values were out of the acceptable range, hence these counts are considered to expose consumers’ health to risk. The European Union recommends that the levels of contamination by total bacteria and total coliforms do not exceed 5.0 and 2.5 log CFU/g, respectively [33].

If the total bacteria count is less than 5.0 log CFU/g and the coliforms count is less than 3.5 log CFU/g, the meat could, therefore, be classified as having low risk as far as transmission of pathogenic bacteria to consumers is concerned [34]. Therefore, the lower level for total bacteria counts and coliform counts from carcasses that were delivered from the LTA, is within the following stipulated range. The counts for beef, mutton and pork were 4.0, 4.9 and 2.9 log CFU/cm^2^, respectively, for the total bacteria counts, and coliform counts were 2.3, 2.6 and 0.7 log CFU/cm^2^, respectively. Therefore, compared to the commercial abattoir, the levels of microbial contamination from carcasses that were delivered from the LTA at the loading stage were low. These results were not expected as the HTA is expected to operate according to standards of hygiene. These findings can best be explained by the lack of a microbiological monitoring programme [4] to determine the hygienic status of the carcass after slaughter and before transportation to the supply points in South African abattoirs. During carcass inspection, only the physiological status of the carcass is assessed. Therefore, risk factors that contribute to the high microbial load in meat are not considered. The few abattoirs that perform microbial assessment do not apply it as part of their daily assessment, and the Department of Veterinary Public Health does it only on a monthly basis during audits. The guidelines used to assess the levels of contamination are based on European standards, and the supermarket chains that perform the microbial assessment on meat products use their standards. These standards were crafted based on European environmental temperatures, which may differ from the temperatures in the southern African region. Such a situation shows a lack of adequate regulations related to meat safety, particularly the Meat Safety Act of 2000. Abattoirs are regulated by this law to ensure good standards of hygiene. Therefore, there is a need to introduce a microbiological assessment approach in abattoirs that can be made part of their daily practices, and the South African government should set up standards that will be mandatory for meat handlers at the abattoirs to be more conscious when handling carcasses.

The lower levels observed from the LTA could be influenced by no cross-contamination of microbes between carcasses from different species since the slaughter of different types of animals was reported to be performed on different days and delivered on the same day after slaughtering [35]. Therefore, their storage period at the abattoir was shorter compared to the commercial abattoir which might have reduced the chances of an increase in the microbial load on the meat. Further, beef had higher levels (*p* < 0.05) of microbial contamination for *E. coli* when compared to pork and mutton in both abattoirs. These results are similar to those in previous reports [36,37]. The higher levels of *E. coli* in beef carcasses can best be explained by cattle being regarded as the main reservoir for *E. coli* O157: H7 [38], since beef and its products are regarded as the main vehicle for transmission of the pathogen [39].

The contamination levels were higher (*p* < 0.05) for *E. coli* on all meat types between the loading and off-loading points from carcasses that were delivered from the HTA. Mutton increased from 1.2 to 3.2 log CFU/cm^2^; followed by pork, with an increase between 1.8 and 2.7 log CFU/cm^2^ and beef, which ranged between 4.7 and 4.9 log CFU/cm^2^. These observations can best be explained by the poor handling of carcasses by workers during loading and off-loading without using handlers’ protective clothing, resulting in cross-contamination of microbes as the carcasses were put on the workers’ shoulders during loading from the chiller rooms to trucks. These observations could also be associated with temperature fluctuations during transportation [40], as doors, when off-loading, would be open for quite some time at each supply point, increasing the temperature inside the trucks.

There was a significant decrease in the microbial load at the display point for the three meat types. The decrease for mutton was 1.2 log CFU/cm^2^, followed by beef with 1.8 log CFU/cm^2^ and pork with 2.5 log CFU/cm^2^. These results contradict the theory [29], whereby retail shop meat is expected to contain a higher microbial load because of the large amount of exposed surface area, with more readily available water, nutrients, and greater oxygen penetration. The higher levels of contamination (*p* < 0.05) for TBC and CC between the loading and off-loading points in the LTA could be associated with handling during transportation [41], as the trucks that were used to deliver the carcasses were just ordinary vans with no refrigerators. Transportation of products to retail outlets and storage is the most common weak link in the chain [25,42,43].

*Staphylococci* are natural flora of the skin and mucous membranes of animals and humans that can cause meat contamination [44]. *Staphylococcus aureus* presence indicates the poor sanitary quality of abattoirs and retail outlets [45]. In the present study, *S. aureus* was not detected at all in samples gathered from the HTA. *Staphylococcus aureus*-positive samples were detected from the LTA on pork carcasses from loading to the display point. *Staphylococcus aureus* was not detected in mutton at the loading and off-loading stages but was observed in small levels (0.5 log CFU/cm^2^) at the display point. These results are similar to those observed in pork carcasses, whereby the levels of contamination for *S. aureus* increased with stage. The presence of *Staphylococcus* on meat in this study can be worrying because certain strains of this bacteria cause food-borne infections [46,47]. These findings mark the urgency of government policymakers in amending the Meat Safety Act of 2000 by introducing meat inspectors that will do meat inspections and prevent the stop of bacteria that cause food-borne infections. The mean microbial load of carcasses at the abattoir was higher (*p* < 0.05) compared to retail meat outlets. These results could best be explained by poor hygienic practices and how the meat was handled in the abattoir and they agree with previous results [48]. However, this contradicts results which reported that the mean values of microbial load of abattoir meat were low (5.04 log CFU/g) compared to butcher shops (5.75 log CFU/g) [29].

### 4.2. Microbial Counts as Affected by Shop Class, Storage Period, Distance and Temperature 

Results on factors affecting the microbial load on meat during distribution from the abattoir to the supply points are shown in Table 2. Shop class, storage period, distance and temperature had an effect (*p* < 0.05) on the microbial load on meat during distribution. Top-class and middle-class shops accepted carcasses that were delivered from the HTA, and the LTA supplied meat to the butcheries. The levels of microbial contamination had an effect (*p* < 0.05) on carcasses that were sold in top- and middle-class shops. In a similar pattern to a past study [49], meat sold from middle-class shops was observed to have higher levels of microbial contamination for TBC, CC and *E. coli* compared to meat sold from high-class shops. The levels of total bacteria count on meat from high-class shops fell within the acceptable range (4.8 log CFU/cm^2^), implying that the meat purchased by consumers from these shops was safe for consumption. Such shows that hygienic practices by meat handlers from top-class shops are followed as the butchery managers had indicated that most of their workers are trained in meat handling. Furthermore, a microbiological assessment of carcasses is carried out before the display of meat is performed to check if the meat is still in good condition, although this is not done daily. Nevertheless, it is a well-known phenomenon that consumers do not consider the microbiological quality when purchasing meat; hence, they cannot tell the risk of incurring a food-borne illness at the time of purchase or consumption of a food item, because the extent of microbial contamination or the level of chemical residues cannot be observed [50]. Consumers use their senses in the description of safe food and feel that food that looks or smells bad should not be consumed [51]. These findings agree with previous reports [52,53].

Carcasses that were delivered to butcheries had the highest levels of microbial contamination for TBC (6.1 log CFU/cm^2^) and CC (4.1 log CFU/cm^2^). These observations could be influenced by improper handling or inadequate storage and display conditions during sales. Such could also be explained by the inconsistent use of disinfectants in work areas, meat handling rooms, and display trays [54]. Another study [32] reported that butcher men lack knowledge of disinfecting and sanitizing, they clean their shops once every 24 h with detergent and water, which is not enough to maintain a hygienic environment in the butchery. Regular cleaning and disinfecting of the retail outlets is important since it helps reduce microbial contamination, making the meat sold in these outlets unsafe for human consumption [32]. It has been highlighted that these microbial groups are safety indicators; therefore, the presence of high counts may indicate the possible presence of pathogens [6].

The storage period of carcasses at different abattoirs had an effect (*p* < 0.05) on the contamination loads. Carcasses that were stored at the HTA after being taken out of the chiller room for less than an hour had a TBC of 6.8 log CFU/cm^2^ compared to carcasses from the LTA, which had fewer counts (4.3 log CFU/cm^2^). The high microbial load on carcasses from the HTA could have resulted from changes in temperature after the carcasses were taken out of the chiller and the duration period before they were loaded into the trucks, which could have led to the multiplication of the microbes. A sudden decrease in the microbial load on carcasses that were kept for 24 h was observed and could have been influenced by the storage of carcasses in chiller rooms, where cold temperature inhibits the growth of microorganisms on meat. A drastic increase in microbial load on carcasses that were delivered to the HTA after 48 h (6.9 log CFU/cm^2^) and 72 h (7.2 log CFU/cm^2^) was observed. A sudden increase in the microbial load on carcasses from the LTA for a storage period of 48 h was observed (5.3 log CFU/cm^2^). Such evidence shows that the longer the storage period of carcasses in an abattoir, the greater the chance of microbial contamination levels increasing. Therefore, meat handlers at the abattoirs would be advised not to keep carcasses for more than 48 h as a strategy for avoiding the growth of microbes and ensuring that safe meat is delivered to consumers [55].

The distance covered by the delivery vehicles when transporting the meat to the supply points was found to have a significant impact on the microbial load developing on the meat. The microbial contamination at the HTA at zero distance for TBC, CC and *E. coli* was 6.9, 5.0 and 2.4 log CFU/cm^2^, respectively. A decrease was observed when the carcasses were transported to the supply point after 1.8 km (5.9, 3.9 and 2.2 log CFU/cm^2^). When carcasses were off-loaded in a retail shop after 2 h, another increase in the microbial load was observed (7.8, 5.6 and 4.7 log CFU/cm^2^) and after 25 km, the microbial counts were between (7.0, 4.9 and 3.0 log CFU/cm^2^). The increase in the microbial load between carcasses that were off-loaded after 1.8 and 2 km could have resulted from the accumulation of air during the off-loading of carcasses since the truck doors at 1.8 km were open for quite some time during off-loading; hence, a slight increase in the temperature of the refrigerated truck was observed. It is a known phenomenon that if the storage temperatures are not properly monitored, meat contamination or multiplication of bacteria in meat may occur, which results in economic losses [56,57]. Therefore, this could explain the increase of the microbial load on carcasses that were delivered at 2 km since they were loaded in the same truck.

The temperature had an effect (*p* < 0.05) on the microbial load of carcasses after slaughter and during transportation. In the LTA, there was no chiller room for storing carcasses after slaughter, and delivery vehicles used for transporting carcasses to the butchery where ordinary trucks with no chiller, TBC and CC ranged between 3.2 and 0.9 log CFU/cm^2^, respectively, when the daily average temperature was 18 °C. Nevertheless, *E. coli* was not detected in these carcasses at this temperature. Microbial contamination for TBC, CC and *E. coli* ranged between 3.9, 1.9 and 0.2 CFU/cm^2^ when daily average temperatures were 20 °C. Microbial counts were 4.9, 2.8 and 0.5 log CFU/cm^2^ at 22 °C. Temperature fluctuations in trucks from the HTA were between 5 and 7 °C. The normal temperature used to keep the carcasses in the trucks was 7 °C; however, at the off-loading points, an increase of 6 and 5 °C was observed.

The Pearson correlation analysis suggests that distance and meat type did not affect the microbial load on meat (see Table 3). However, a significant negative correlation between distance and distribution stage was observed, although a significant positive correlation (*p* < 0.05) with microbial contaminants (TBC, CC and *E. coli*) was also observed. The storage period also had a significant negative correlation with the microbial contaminants on meat, although no relationship with *S. aureus* was observed. These findings suggest that the longer the distance and the storage period, the higher the level of microbial contamination in the meat. Temperature also had a positive correlation (*p* < 0.05) with the distribution stage and shop class. This can be related to the fact that different shops used different temperatures for storage, as well as the fluctuating temperatures experienced at different stages and when distributing the carcasses [58].

## 5. Conclusions

Meat from both HTA and LTA contained higher levels of contamination by *Escherichia coli,* total bacteria and coliform counts, as they were observed to have exceeded the acceptable limits. The contamination levels obtained were even higher on meat from the HTA throughout the distribution chain compared to the LTA. Carcass contamination between loading and offloading points was higher. Additionally, the bacterial counts of meat samples sold through middle-class shops and butcheries were higher compared to top-class shops. Therefore, it was concluded that there is a need for meat handlers and retailers to rigidly enforce standard hygienic practices throughout the distribution chain. In addition, microbiological assessment of carcasses in abattoirs and during marketing should be introduced to ensure meat safety. However, there will be a need to devise efficient methods to practically perform such practices on a daily basis.

## Figures and Tables

**Table 1:** Least square mean values (±SE) for microbial counts per meat type at different stages of the distribution chain in each abattoir.

			HTA				LTA		
		Microbial Contaminants				Microbial Contaminants			
Stage	Meat Type	Total Bacteria Count (log CFU/cm^2^)	Coliform Count (log CFU/cm^2^)	Presumptive *E. coli* (log CFU/cm^2^)	*S. aureus* (log CFU/cm^2^)	Total Bacteria Count (log CFU/cm^2^)	Coliform Count (log CFU/cm^2^)	Presumptive *E. coli* (log CFU/cm^2^)	*S. aureus* (log CFU/cm^2^)
Loading	Mutton	7.2 ± 0.16 ^b^	4.2 ± 0.39 ^a^	1.2 ± 0.34 ^a^	0.0 ± 0.00 ^a^	4.9 ± 0.42 ^c^	2.6 ± 0.34 ^b^	0.2 ± 0.15 ^a^	0.0 ± 0.00 ^a^
	Pork	5.9 ± 0.35 ^a^	3.9 ± 0.29 ^a^	1.8 ± 0.44 ^a^	0.0 ± 0.00 ^a^	2.9 ± 0.20 ^a^	0.7 ± 0.29 ^a^	0.2 ± 0.14 ^a^	0.3 ± 0.24 ^b^
	Beef	7.7 ± 0.17 ^b^	5.8 ± 0.30 ^b^	4.7 ± 0.26 ^c^	0.0 ± 0.00 ^a^	4.0 ± 0.39 ^b^	2.3 ± 0.28 ^b^	0.4 ± 0.23 ^b^	0.0 ± 0.00 ^a^
Offloading	Mutton	6.1 ± 0.36 ^a^	3.6 ± 0.30 ^a^	3.2 ± 0.92 ^b^	0.0 ± 0.00 ^a^	4.80 ± 0.45 ^b^	2.6 ± 0.30 ^b^	0.1 ± 0.14 ^b^	0.0 ± 0.00 ^a^
	Pork	6.5 ± 0.40 ^a^	4.8 ± 0.41 ^b^	2.7 ± 0.43 ^b^	0.0 ± 0.00 ^a^	3.2 ± 0.23 ^a^	0.9 ± 0.27 ^a^	0.0 ± 0.00 ^a^	0.4 ± 0.23 ^b^
	Beef	7.7 ± 0.20 ^b^	5.8 ± 0.22 ^b^	4.9 ± 0.16 ^c^	0.0 ± 0.00 ^a^	5.0 ± 0.27 ^b^	2.7 ± 0.48 ^b^	0.7 ± 0.27 ^c^	0.0 ± 0.00 ^a^
Display	Mutton	5.4 ± 0.35 ^a^	4.1 ± 0.35 ^a^	1.2 ± 0.49 ^a^	0.0 ± 0.00 ^a^	6.1 ± 0.40 ^b^	5.2 ± 0.36 ^b^	0.1 ± 0.15 ^b^	0.5 ± 0.32 ^b^
	Pork	7.0 ± 0.37 ^b^	4.5 ± 0.25 ^b^	2.5 ± 0.56 ^b^	0.0 ± 0.00 ^a^	4.7 ± 0.35 ^a^	2.3 ± 0.31 ^a^	0.0 ± 0.00 ^a^	0.4 ± 0.35 ^b^
	Beef	6.7 ± 0.40 ^ab^	6.1 ± 0.36 ^c^	1.8 ± 0.68 ^a^	0.0 ± 0.00 ^a^	4.8 ± 0.47 ^a^	4.8 ± 0.66 ^b^	0.1 ± 0.17 ^b^	0.0 ± 0.00 ^a^

^abc^ Means in the same column for beef, pork and mutton (within each stage) with different superscripts are significantly different (*p* < 0.05).

**Table 2:** Least square mean values (±SE) for beef, pork and mutton carcass contamination as affected by shop class, storage period and distance.

Description	Total Bacteria Count (log CFU/cm^2^)	Coliform Count (log CFU/cm^2^)	Presumptive *E. coli* (log CFU/cm^2^)	*S. aureus* (log CFU/cm^2^)
Class				
Top-class shop	4.8 ± 0.61 ^a^	2.9 ± 0.61 ^a^	1.9 ± 0.67 ^a^	0.1 ± 0.23 ^a^
Middle-class shop	5.7 ± 0.58 ^ab^	4.2 ± 0.57 ^b^	2.3 ± 0.63 ^b^	0.0 ± 0.21 ^a^
Butchery	6.1 ± 0.61 ^b^	4.1 ± 0.60 ^b^	1.6 ± 0.66 ^a^	0.1 ± 0.22 ^a^
Storage period				
Commercial				
>1 h	6.8 ± 0.22 ^b^	4.7 ± 1.44 ^a^	3.5 ± 0.36 ^c^	0.0 ± 0.000 ^a^
24 h	6.1 ± 0.27 ^a^	4.6 ± 0.24 ^a^	1.5 ± 0.35 ^a^	0.0 ± 0.000 ^a^
48 h	6.9 ± 0.27 ^c^	5.1 ± 0.38 ^b^	2.3 ± 0.53 ^ab^	0.0 ± 0.000 ^a^
72 h	7.2 ± 0.23 ^b^	4.7 ± 0.28 ^a^	2.9 ± 0.39 ^b^	0.0 ± 0.000 ^a^
Communal				
>1 h	4.3 ± 0.23 ^b^	2.1 ± 0.23 ^a^	0.3 ± 0.11 ^b^	0.1 ± 0.08 ^a^
2 h	3.9 ± 0.25 ^a^	1.9 ± 0.22 ^a^	0.3 ± 0.10 ^b^	0.1 ± 0.07 ^a^
48 h	5.3 ± 0.26 ^c^	3.9 ± 0.33 ^b^	0.1 ± 0.07 ^a^	0.3 ± 0.16 ^b^
Distance				
Commercial				
0 km	6.9 ± 0.17 ^b^	5.0 ± 0.21 ^b^	2.4 ± 0.29 ^a^	0.0 ± 0.000 ^a^
1.8 km	5.9 ± 0.23 ^a^	3.9 ± 1.14 ^a^	2.2 ± 0.46 ^a^	0.0 ± 0.000 ^a^
2 km	7.8 ± 0.23 ^c^	5.6 ± 0.22 ^c^	4.7 ± 0.19 ^c^	0.0 ± 0.000 ^a^
25 km	7.0 ± 0.31 ^b^	4.9 ± 0.29 ^b^	3.0 ± 0.45 ^b^	0.0 ± 0.000 ^a^
Communal				
0 km	4.6 ± 0.19 ^b^	2.9 ± 0.23 ^b^	0.2 ± 0.06 ^a^	0.2 ± 0.09 ^a^
1.5 km	4.3 ± 0.23 ^a^	2.1 ± 0.24 ^a^	0.3 ± 0.11 ^a^	0.1 ± 0.08 ^a^

^abc^ Means in the same column for beef, pork and mutton with different superscripts are significantly different (*p* < 0.05).

**Table 3:** Pearson correlation analysis between factors affecting bacterial load on meat and microbial contaminants.

	Temperature	Meat Type	Stage	Shop Class	Storage Period	TBC	CC	PEC	*S. aureus*
Distance	−0.19 **	0.01	−0.19 **	−0.09	−0.01	0.24 **	0.18 **	0.25 ***	−0.06
Temperature		−0.01	0.34 ***	0.55 ***	−0.43 ***	−0.46 ***	−0.53 ***	−0.31 ***	0.01
Meat Type			−0.01	0.01	0.12	0.05	0.18 **	0.22 ***	−0.06
Stage				0.80 ***	−0.34 ***	−0.49 ***	−0.36 ***	−0.57 ***	0.21 *
Shop Class					−0.31 ***	−0.52 ***	−0.45 ***	−0.53 ***	0.17 *
Storage period						0.37 ***	0.43 ***	0.22 **	−0.05
TBC							0.75 ***	0.57 ***	−0.07
CC								0.54 ***	−0.13 *
PEC									−0.13 *

Significantly correlated at * *p* < 0.05; ** *p* < 0.01, *** *p* < 0.001. TBC = total bacteria count; CC = coliform count; PEC = presumptive *E. coli.*

## Data Availability

The datasets used during the current study are available from the corresponding author on reasonable request.
